# A Novel Low-Cost Bio-Sorbent Prepared from Crisp Persimmon Peel by Low-Temperature Pyrolysis for Adsorption of Organic Dyes

**DOI:** 10.3390/molecules27165160

**Published:** 2022-08-12

**Authors:** Lu-Qing Xie, Xin-Yu Jiang, Jin-Gang Yu

**Affiliations:** College of Chemistry and Chemical Engineering, Central South University, Changsha 410083, China

**Keywords:** crisp persimmon peel, vacuum pyrolysis, low-cost adsorbent, selective adsorption, low-temperature pyrolysis, organic dyes

## Abstract

In order to properly reuse food waste and remove various contaminants from wastewater, the development of green, sustainable and clean technologies has demonstrated potential in the efficient inhibition of secondary pollution to the environment. In this study, an economical and green method was used to prepare biochar from crisp persimmon peel (CPP) using flash-vacuum pyrolysis at different temperatures (200–700 °C; referred to as CPP200–CPP700). CPP200 has high polarity, low aromaticity and high oxygen-containing functional groups that exhibit superior MB adsorption capabilities. CPP200 that was prepared at a relatively low temperature of 200 °C exhibited a high adsorption capacity of 59.72 mg/g toward methylene blue (MB), which was relatively higher than that for alizarin yellow R (4.05 mg/g) and neutral red (39.08 mg/g), indicating that CPP200 possesses a higher adsorption selectivity for cationic dyes. Kinetics investigation revealed that the kinetic data of CPP200 for the adsorption of MB was better fitted by a linear pseudo-second-order model. Isothermal studies indicated that the linear Langmuir model was more suitable for describing the adsorption process. The adsorption thermodynamics illustrated that the adsorption of MB onto CPP200 was spontaneous and endothermic. EDS and IR analyses of CPP200 for both pre- and post-adsorption of MB showed that electrostatic interactions between oxygen-containing groups on biochar and target MB dominated the adsorption procedure, in addition to hydrogen bonding interactions. Reusability tests confirmed the excellent regeneration characteristics of CPP200, indicating that CPP200 may be used as a green, sustainable, highly efficient and recyclable adsorbent for the selective removal of cationic organic dyes.

## 1. Introduction

The discharge of dye-containing wastewater into water bodies causes serious water pollution. For examples, pollutant dyes such as methylene blue (MB), alizarin yellow R (AYR) and neutral red (NR) have been widely used in food, textiles, cosmetics, papermaking, coatings, printing, pharmaceuticals and other industries [1]. Due to their long-term persistence, dye-containing wastewater hinders light penetration, weakens photosynthesis of aquatic plants and algae, resulting in reductions in dissolved oxygen levels and leading to the eutrophication of waters [2]. Furthermore, organic dyes with their complex aromatic structures have been associated with carcinogenesis in aquatic animals [3]. The existence of organic dyes in aquatic environments is highly dangerous to aquatic animals and plants [4]. In addition, the bioaccumulation of organic dyes can cause severe allergies and skin irritations to human beings [5]. Therefore, it is necessary to develop facile and reliable approaches for the safe and efficient removal of these harmful pollutants from dye-containing wastewaters before they are discharged into environmental waters. Various physio-chemical technologies used for the treatment of organic dyes, such as chemical oxidation, electrochemical degradation, adsorption and advanced photocatalytic degradation, etc., have been extensively investigated and implemented [6,7,8,9]. Among these approaches, adsorption has attracted extensive attention for its simple operation and cost-saving properties [10]. Nevertheless, efficient and economical adsorbents with good handling properties are always required [11]. In particular, natural product-based adsorbents that are derived from renewable and biodegradable resources are more attractive because of their environmentally friendly, inexpensive and sustainable features [12]. Biochars (BCs) have been considered one of the most preferred adsorbents for contaminant removal [13], and they have attracted much attention because of their wide and low-cost raw sources available [14]. Many previous studies reported the preparation of biomass-based adsorbents using various methods, such as covalent grafting of functional organic units, doping heteroatoms or metal elements [15,16,17]. BCs prepared by direct pyrolysis have been also widely investigated. For instance, BCs obtained from pyrolysis of persimmon peel at 700 °C could be applied to the adsorption of MB [18]. Currently, most pyrolysis experiments have been conducted at relatively higher temperatures, which may result in secondary pollution to the environment. The pyrolysis of biomass is often responsible for the production of toxic substances such as dioxins and polycyclic aromatic hydrocarbons (PAHs). Dioxins and PAHs are the main byproducts of the thermal decomposition of lignocellulose, and the discharge of these dioxins and PAHs increases as the pyrolysis temperature increases [19]. There has been some concern over the emission of PAHs, since they are associated with human teratogenesis, cancer or mutations due to their bio-accumulative effects [20]. A decrease in the discharge of PAHs and dioxins can be achieved by using lower pyrolysis temperatures [21,22]. Definitely, the discharge of some toxic volatile components may occur even under low-temperature pyrolysis conditions. Importantly, the exhaust gases can be effectively eliminated by using a vacuum pump together with a gas cleaning system [23]. Therefore, low-temperature, flash-vacuum pyrolysis technology appears to be relatively more environmentally friendly. Interestingly, the reservation of heteroatomic substances in BCs may be beneficial to its highly-efficient adsorption [24].

In 2017, the world produced about 5.7 million tons of persimmons, and the yield of persimmon production in Asia was 5.1 million tons, confirming the significant production of persimmons and hence the possible production of large quantities of persimmon peel (PP) [25]. Moreover, the main ingredients of persimmons, such as carotenoids, tannins, cellulose, lignin and fiber, have granted its antiradical and antibacterial activities [26]. In particular, previous research has also indicated that tannins could be used to remove various contaminants in aqueous solutions [27]. The high concentration of tannins in persimmons has implied a strong possibility of using crisp persimmon peel (CPP)-derived BCs as efficient adsorbents.

In this study, CPP-derived BCs were prepared by a low-temperature, flash-vacuum pyrolysis procedure. We were also delighted to find that CPP-based BC prepared at a low temperature of 200 °C (CPP200) exhibited relatively higher adsorption capacities toward methylene blue (MB) than BC prepared at higher temperatures. Moreover, CPP200 showed a higher adsorption selectivity toward cationic dyes (MB) than anionic dyes (alizarine yellow R, AYR) and neutral dyes (neutral red, NR). In order to explore the adsorption mechanism, the changes in elemental compositions, heteroatomic functional groups and microstructures of CPP-derived BCs at different temperatures were analyzed using Fourier transform-infrared (FT-IR) spectroscopy, scanning electron microscopy with energy dispersive X-ray spectrometry (SEM/EDS), and the elemental mapping of CPP200 pre- and post- adsorption of MB. In addition, batch adsorption experiments were carried out in order to explore the adsorption kinetics, thermodynamics, selectivity, reusability and adsorption efficiency of CPP200 toward MB with actual water samples. The low-temperature, flash-vacuum pyrolysis method has shown energy-saving and environmentally friendly features. Meanwhile, CPP-derived BCs can be rapidly and conveniently produced, and can thus be used as cost-saving adsorbents for future industrial and practical applications. This paper will serve as a useful reference for the further treatment of green resources, help us to understand the sustainable utilization of agricultural and forestry waste, and avoid further contribution to greenhouse effects.

## 2. Materials and Methods

### 2.1. Chemicals

CPPs were torn down from crisp persimmon fruits which were purchased from a local supermarket in Changsha (Hunan Province, China). Methylene blue (MB, 98.5 wt.%) was purchased from Beijing Chemical Factory (Beijing, China). Alizarin yellow R (AYR, 90 wt.%) was obtained from Hunan Xiangzhong Chemical Industry Co., Ltd. (Loudi, China). Neutral red (NR, 98 wt.%) was purchased from Tianjin Fuchen Chemical Reagent Co., Ltd. (Tianjin, China). Hydrochloric acid (HCl) with a purity of 37 wt.% was purchased from Chengdu Colon Chemicals Co., Ltd. (Chengdu, China). Sodium hydroxide (NaOH, 96 wt.%) was acquired from Tianjin Hengxing Chemical Preparation Co., Ltd. (Tianjin, China). Ultrapure water with a resistivity of 18.2 MΩ cm was obtained using a Millipore Milli-Q water purification system (Millipore Trading Co., Ltd., Shanghai, China). All chemicals were of analytical grade and used without further purification.

### 2.2. Preparation of BCs

**Figure 1:**
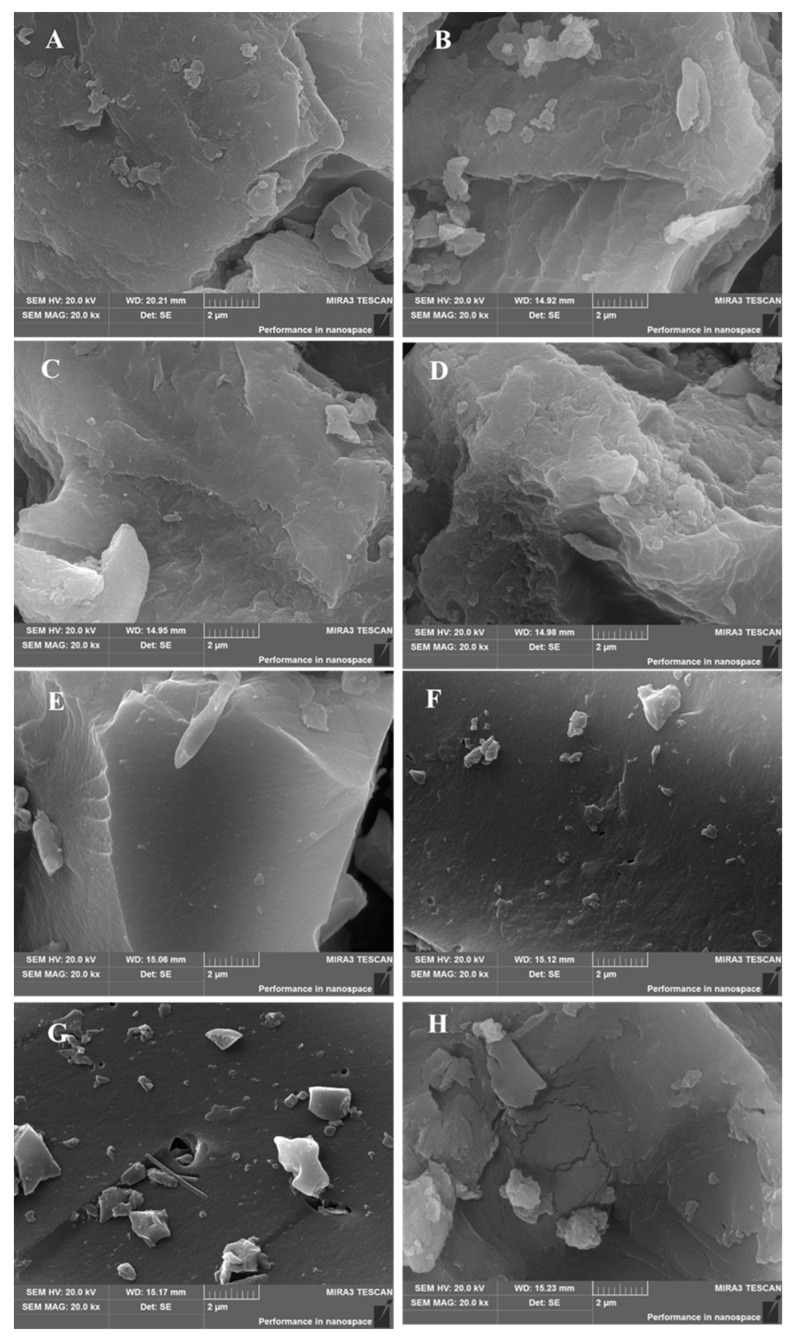
SEM images of BC samples: (**A**) CPP200; (**B**) CPP250; (**C**) CPP300; (**D**) CPP350; (**E**) CPP400; (**F**) CPP450; (**G**) CPP500; (**H**) CPP200, post-adsorption of MB.

CCPs were naturally dried in an indoor environment, then dried in an oven at 150 °C for 30 min. After that, CCPs were rinsed with tap water, then dried in an oven at 120 °C for 1 h. The treated CCPs were cut into pieces, put into a porcelain boat and wrapped in tin foil. The samples were pyrolyzed in a high-temperature vacuum tube furnace (Model: SGM-6810A; Luoyang Sigma Instrument Manufacturing Co., Ltd., Luoyang, China) at different temperatures (200 °C, 250 °C, 300 °C, 350 °C, 400 °C, 450 °C and 500 °C) (Figure 1). The pyrolysis of CPPs was carried out in a horizontal tubular reactor, which was vacuated with an air pump until the pressure was reduced to 0.1 MPa. The pyrolysis was conducted for 160 min (heating time plus residence time) at a heating rate of 5 °C min^−1^. The BC samples obtained at 200 °C, 250 °C, 300 °C, 350 °C, 400 °C, 450 °C and 500 °C were defined as CPP200, CPP250, CPP300, CPP350, CPP400, CPP450 and CPP500, respectively. The obtained BCs were grounded in a mortar for 30 min, washed repeatedly with ultrapure water 2 times and with ethanol 3 times, then dried in an oven at 60 °C until constant weight was achieved before being transferred to a dryer for future use.

### 2.3. Characterization of BC Samples

BCs were characterized using different techniques. The surface morphologies of as-prepared BC samples were observed via SEM (JEOL, JSM-6360LV; Tokyo, Japan), and their elemental percentages [including carbon (C), nitrogen (N), oxygen (O) and sulfur (S)] were analyzed using energy dispersive X-ray spectroscopy (EDS). In order to explain the changes of CPP200 pre- and post- adsorption of MB, the CPP200 after MB adsorption was also analyzed via SEM and element mapping. An FT-IR (Model: Shimadzu IR Prestige 21; Japan) spectrophotometer within the wavenumber range 4000–400 cm^−1^ and a resolution of 4 cm^−1^ was used to observe all functional groups of BC samples as well as the changes in functional groups of CPP200 post-adsorption of various dyes. The specific surface areas (SSAs) of CPP200 and CPP500 were examined using a Kubo X1000 automated surface area analyzer (Beijing Builder Electronic Technology Co., Ltd., Beijing, China); the test accuracy of the instrument was ±1% (≤1 m^2^/g samples: ±1.5%). In order to further confirm the chemical composition of CPP200, X-ray photoelectron spectroscopic (XPS) analysis was carried out on a Perkin Elmer PHI 5000 C ESCA instrument with Al Kα radiation operated at 250 W (Perkin-Elmer, Norwalk, CT, USA). The cation exchange capacity (CEC) of CPP200 was determined according to a simplified forced-exchange method [28]. Briefly, 100.0 mg of BC sample was added into a conical flask; then, 20.0 mL of 0.25 M BaCl_2_ was added in. The mixture was oscillated in a shaker at 200 rpm for 2 h and then filtered. The concentrations of exchangeable ions (Na, Mg, K, Ca, Al, Mn, Zn) in the filtrate were detected using inductively coupled plasma-optical emission spectrometry (ICP-OES). CEC was calculated by the sum of all cations in the BC sample (cmol_c_/g).

### 2.4. Batch Adsorption Studies

The adsorption experiments were carried out by adding 5.0 mg of BC into a conical flask (100 mL) that contained 20.0 mL of dye solution with the desired concentrations. The adsorption of the dye solution both pre- and post-adsorption was measured using a 752 UV-Vis spectrophotometer (Shanghai Jinghua Technology Instrument Co., Ltd., Shanghai, China) in a wavelength-specific manner (664 nm for MB, 370 nm for AYR and 542 nm for NR) at room temperature, and dye concentrations were calculated through the calibration equations.

The adsorption of MB onto BCs prepared at different temperatures was measured at 25 °C for 90 min. The optimization of adsorption conditions was carried out using CPP200, which exhibited the largest adsorption capacity. Effects of contact time, temperature, initial MB concentration, dosage of adsorbent and solution pH value on the removal of MB by CPP200 were investigated in detail. The effect of contact time (5 min, 10 min, 15 min, 30 min, 60 min, 90 min, 120 min, 150 min) on CPP200 (5.0 mg) toward 20.0 mL of MB (50 mg/L, pH = 7.32) was observed at 25 °C. Effects of initial MB concentrations (10 mg/L, 20 mg/L, 30 mg/L, 40 mg/L, 50 mg/L) and temperatures (25 °C, 30 °C, 35 °C, 40 °C, 45 °C) on the adsorption capacity of CPP200 for MB were investigated (equilibrium time = 90 min, 5.0 mg of CPP200, 20.0 mL MB, initial pH = 7.32). Effects of solution pH values (3.33, 4.34, 5.35, 6.30, 7.32, 8.34, 9.36) on CPP200 for MB solution (20.0 mL, 50 mg/L) at 25 °C were observed over periods of 90 min. Aqueous hydrochloric acid (HCl, 0.1 mol/L) and sodium hydroxide (NaOH, 0.1 mol/L) solutions were used for pH adjustment. The solution pH values were measured through a pH meter (Model: PHS-3C; Shanghai INESA Scientific Instrument Chemical Co., Ltd., Shanghai, China). Effects of adsorbent dosages (5.0 mg, 10.0 mg, 15.0 mg, 20.0 mg, 25.0 mg) for MB (20.0 mL, 50 mg/L, pH = 7.32) at 25 °C were examined in periods of 90 min. For the regeneration experiments, 50.0 mg of CPP200 was shaken in 20.0 mL of MB (10 mg/L) at 25 °C for 90 min under oscillatory conditions, and the desorption of MB from CPP200 was implemented using 40.0 mL of HCl (0.1 mol/L).

Batch adsorption experiments were conducted, and the results of three parallel experimental studies were recorded. Equation (1) was applied to calculate the adsorption capacity of BCs, and the adsorption rate (R, %) was calculated according to Equation (2):

(1) $$q_{e}=\frac{(C_{0}-C_{e})\times V}{m}$$

(2) $$R=\frac{C_{0}-C_{e}}{C_{0}}\times 100\%$$

where *C*_0_ (mg/L) is the initial MB concentration; *C_e_* (mg/L) is the equilibrium concentration of MB; *V* (L) and *m* (g) are the volume of MB solution and the mass of BC added to the solution, respectively; *q_e_* (mg/g) represents the equilibrium adsorption capacity of the BC.

## 3. Results and Discussion

### 3.1. Characterization of BCs

**Figure 2:**
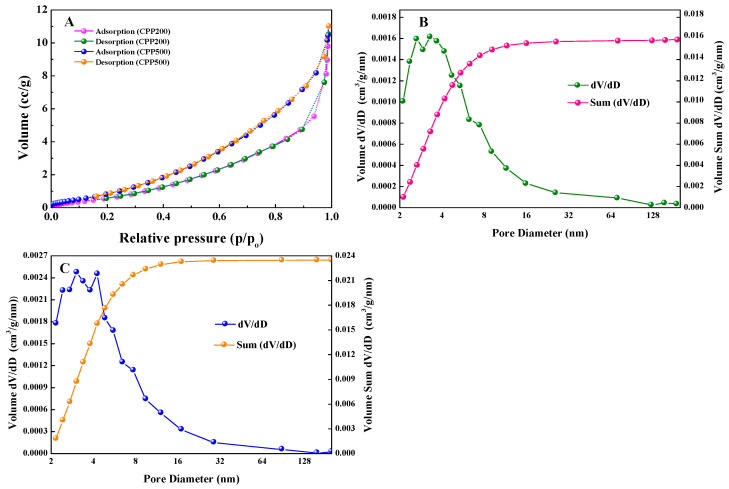
(**A**) N_2_ adsorption/desorption isotherms for CPP200 and CPP500; (**B**) BJH pore volume distribution of CPP200; (**C**) BJH pore volume distribution of CPP500.

The effects of pyrolysis temperature on the physicochemical properties (basic composition, structural properties and surface functional groups) of BCs were studied. The surface morphologies of BCs were observed via SEM (Figure 1A–G). Obviously, BC samples after grinding all show irregular morphologies. Irregular-shaped protrusions were observed on the surfaces of CPP200, CPP250, CPP300 and CPP350 that were pyrolyzed at lower temperatures. When the pyrolysis temperatures were higher, BCs’ surfaces became smoother. The micropores of the samples after grinding were further detected using BET analysis. As shown in Figure 2A, the specific surface area (SSA) and the total pore volume (TPV) of CPP200 were calculated to be 1.1931 m^2^/g and 0.013629 cm^3^/g, respectively. Both the SSA and TPV of BCs increased as pyrolysis temperature increased, which were 1.6900 m^2^/g and 0.017045 cm^3^/g for CPP500, respectively. Obviously, the TPV became larger with an increase in pyrolysis temperature. The pore sizes and size distributions present in the BCs determined from low-temperature N_2_ adsorption/desorption measurements were also affected, indicating that the thermal decomposition of more volatile components occurred under higher temperatures, and high levels of carbonization were implemented (Figure 2B,C).

SEM images of CPP200 post-adsorption of MB are presented in Figure 1H. CPP200 seems to thicken as a result of MB adsorption onto the carbonaceous surface. The observed CPP200-MB with varied thickness might be related to an uneven distribution of polar functional groups, such as -COOH and -OH, onto the surface of CPP200.

**Figure 3:**
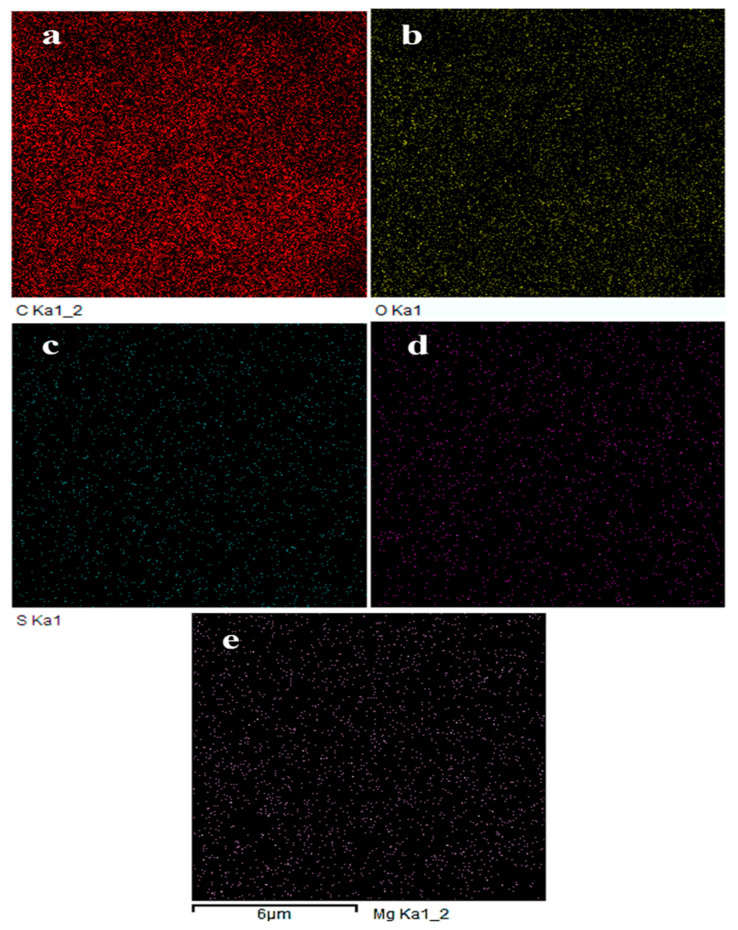
Elemental mapping of CPP200: (**a**) C; (**b**) O; (**c**) S; (**d**) Ca; and (**e**) Mg.

The elemental compositions of BCs were also analyzed (Table 1). The higher the pyrolysis temperature used, the more the volatile molecules that were released. That is, higher levels of carbonization occurred at higher pyrolysis temperatures. In the pyrolysis temperature range of 200–350 °C, the atomic ratio of carbon (C) in BCs remained stable at lower levels (61.09~71.14 at.%), which was much lower than those prepared at higher pyrolysis temperatures in the range of 400–500 °C. In contrast, the atomic ratios of oxygen (O) (25.54~37.35 at.%) of BCs that were prepared at lower temperatures were higher than those (11.30~15.63 at.%) prepared at higher temperatures. Due to the homogeneity of raw materials, the atomic ratio of sulfur (S) varied without regularity (Figure 3a–c). Alkali metal elements could be detected in BC samples (Figure 3d,e). The CEC value of CPP200 was determined by the sum of all cations to be 0.0218 cmol_c_/g, and the contents of alkali metal elements decreased significantly after the adsorption process, confirming that the water-soluble salts of BCs would be released [29]. The newly appeared nitrogen (N) content as well as the increased S content in the sample after adsorption of MB confirmed the successful adsorption of N- and S-containing MB.

**Figure 4:**
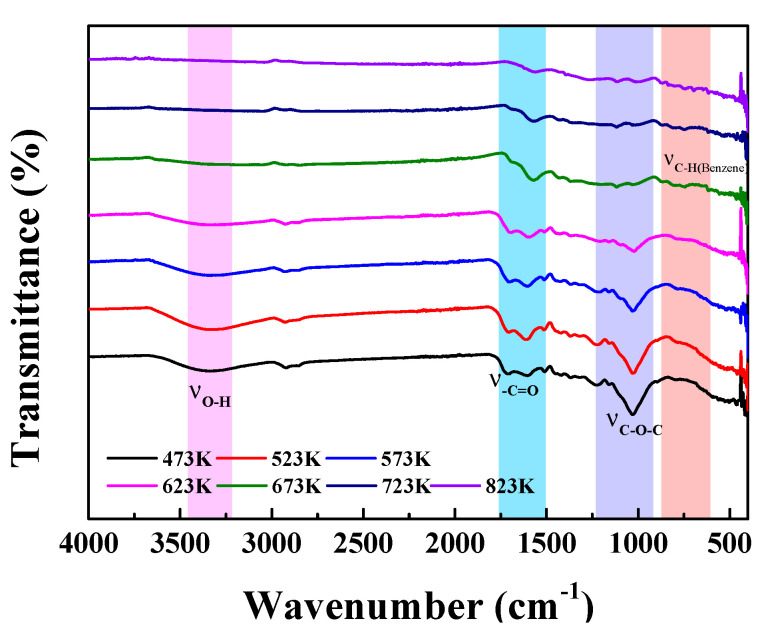
FT−IR spectra of BCs prepared using different pyrolysis temperatures.

The FT-IR spectra of BCs are shown in Figure 4. CPP200–CPP350 produced at low temperatures preserved most of carbohydrate components of the raw biomass. In the pyrolysis temperature range of 200–350 °C, no obvious changes could be observed in the FT-IR spectra. The peaks at 3337 cm^−1^ correspond to the stretching vibration of O–H [30]; the absorption bands at 2925 and 2846 cm^−1^ correspond to the C–H stretching vibrations of alkyl groups [31]; the stretching vibrations of –C=O of aldehydes or esters appear at various wave numbers in the range of 1511~1712 cm^−1^ [32]; the skeletal vibrations of the O–H plane could be observed at 1456 cm^−1^; the peaks appearing at 1317~1368 cm^−1^ and 1030~1225 cm^−1^ could be attributed to the C–O and C–O–C stretching vibrations, respectively [33]. The FT-IR results suggest that BCs retain a large number of original components, such as fatty acids, sugars, lignin and polar cellulose-containing molecules [34]. As the temperature increased, thermal pyrolysis occurred in the absence of oxygen, and volatile ingredients would be produced; the content of aromatic structures would appear and gradually increase. Other functional groups such as hydroxyl, alkyl and C–O–C groups were gradually pyrolyzed, and the peaks’ heights decreased [35]. When the pyrolysis temperature reached above 400 °C, the aromatic structures became the dominant functional groups [36]. The bands in the wave number range of 1570~1688 cm^−1^ and also at 1433 cm^−1^ could be assigned to aromatic skeletal vibrations, and the bands in the wave number range of 619~873 cm^−1^ correspond to in-plane bending vibrations of aromatic hydrocarbons, indicating that BCs obtained at higher pyrolysis temperatures were highly carbonized [37]. Obviously, C content in BCs increased while O content decreased with an increase in pyrolysis temperature, and this was consistent with the results of elemental analyses, again confirming that the higher carbonization of BCs occurred at higher temperatures.

**Figure 5:**
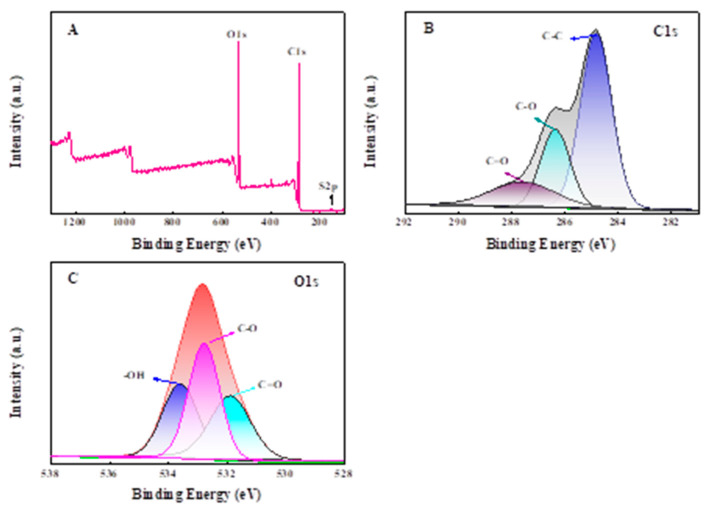
XPS analysis: (**A**) survey XPS spectra of CPP200; and XPS-peak-differentation-imitating analyses of C1s (**B**) and O1s (**C**).

The elemental compositions and surface groups of CPP2000 were characterized by XPS (Figure 5). Obviously, CPP200 mainly contains C (51.57 wt.%), O (45.67 wt.%) and a small amount of S (0.12 wt.%) (Figure 5A), a finding that is consistent with the results of elemental analysis (Table 1). XPS-peak-differentiation-imitating analyses of C1s and O1s were also simulated. As shown in Figure 5B,C, the peak for the C1s can be deconvoluted into three components at 284.8 eV, 286.4 eV and 287.6 eV, which may be attributed to sp^3^ C–C (58.40%), C–O (24.89%) and C=O (16.72%) bonds, respectively [30]. The deconvolution of the peak for the O1s confirmed the existence of C=O (28.91%), C-O (39.57%) and -OH (31.52%) species at binding energies of 533. 6 eV, 532.7 eV and 531.9 eV, respectively [38]. The XPS results again indicate that there are a considerable number of oxygen-containing functional groups on the surface of CPP200.

### 3.2. Effect of Pyrolysis Temperature on BCs toward MB Removal

**Figure 6:**
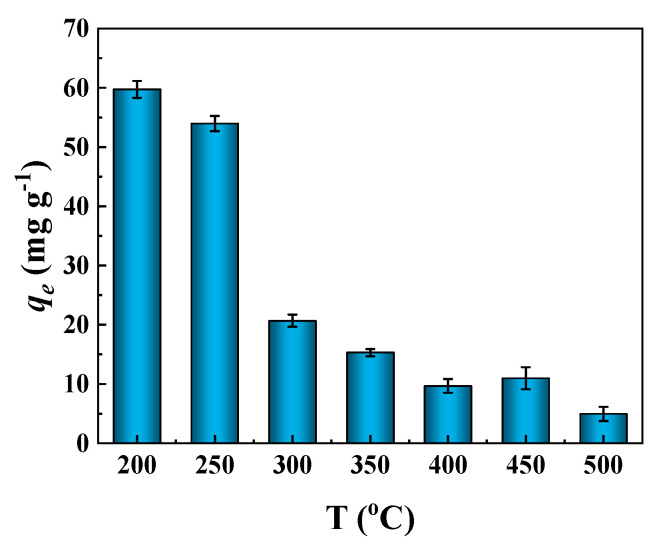
Adsorption of MB by various BCs (CPP200, CPP250, CPP300, CPP350, CPP400, CPP450 and CPP500; MB concentration of 50 mg/L, pH = 7.32, adsorbent dosage of 5.0 mg and contact time of 90 min, T = 25 °C).

The adsorption capacities of BCs derived from CPP toward MB at different temperatures were discussed (Figure 6). Clearly, CPP200 possessed the highest adsorption capacity for MB. Although BET data showed that the SSA and TPV of CPP500 were larger than CPP200, the adsorption capacities of BCs gradually decreased with an increase in temperature, indicating that surface functional groups dominated the adsorption process, and that a decrease in surface functional groups would negatively affect adsorption capacities. The existence of more oxygen-containing groups on the surfaces of BCs could provide more negatively charged sites, which facilitate electrostatic attractions with positively charged MB; hence, higher adsorption capacities could be achieved with these groups [39,40]. The elemental analyses also showed that an increase in pyrolysis temperature would lead to a loss of oxygen-containing groups. A decrease in the O/C ratio would cause a subsequent decrease in surface hydrophilicity and surface polar functional groups, and hence BCs prepared at higher temperatures would become less hydrophilic [41].

### 3.3. Effect of Adsorption Conditions

#### 3.3.1. Effect of Contact Time

**Figure 7:**
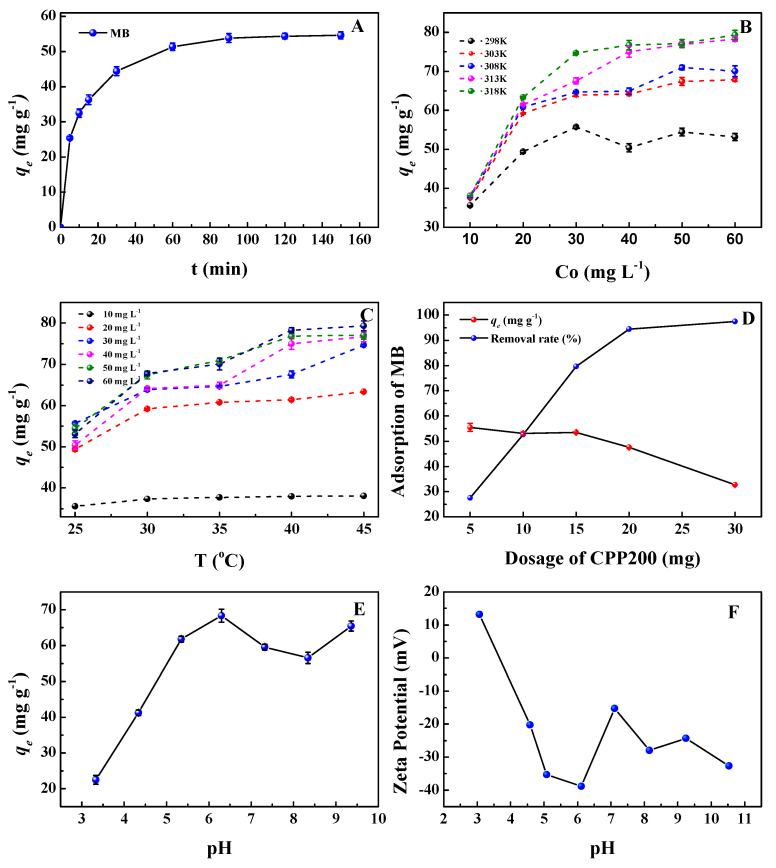
Adsorption properties of CPP200 for MB. (**A**) Effect of contact time (*C*_0_ = 50.0 mg/L, T = 25 °C, pH = 7.32); (**B**,**C**) effects of initial MB concentration and temperature (t = 90 min, pH = 7.32); (**D**) effect of CPP200 dosage (*C_0_* = 50.0 mg/L, t = 90 min, pH = 7.32); (**E**) effect of initial MB pH (*C*_0_ = 50.0 mg/L, t = 90 min, T = 25 °C); (**F**) zeta potential of CPP200 in aqueous solution at different pH values.

The adsorption of MB on CPP200 at different time intervals (0–150 min) was studied (Figure 7A). A two-stage adsorption process could be observed. The initial adsorption corresponded to the availability of the active adsorption sites on the CPP200 surface which became rapidly saturated [42]; the adsorption capacity reached 44.40 mg/g within 30 min. The second stage of establishing the equilibrium of adsorption was slowly achieved over 90 min, with a maximum adsorption capacity of 54.26 mg/g. Therefore, the time for adsorption to reach equilibrium is 90 min.

#### 3.3.2. Effects of Temperature and Initial MB Concentration

The adsorption isotherms of CPP200 toward MB were investigated (Figure 7B). The adsorption of MB increased sharply at low concentrations initially, and then reached a plateau. At low temperature (298 K), the adsorption capacity of CPP200 reached its maximum at an initial MB concentration of 30 mg/L and did not change as the initial concentration was continuously increased. As the temperature increased, the adsorption capacity of CPP200 gradually improved with an increase in initial MB concentration. We proposed that the interactions between MB and CPP200 surface were energy driven, and that higher temperatures were beneficial for the migration of MB to the CPP200 surface, indicating that the adsorption process was endothermic. At higher MB concentrations, the active sites of CPP200 would become rapidly and fully occupied, again confirming that the main adsorption was actually derived from the oxygen-containing functional groups on the surface. In particular, the adsorption capacity of CPP200 did not significantly change as the temperature increased from an initial MB concentration of 10 mg/L, suggesting that the competitive adsorption between MB and water molecules could not be ignored at lower initial MB concentrations (Figure 7C). At an initial MB concentration of 60 mg/L, the maximum adsorption capacity of CPP200 reached 77.11 mg/g at high temperatures (≥313 K) and remained nearly unchanged (time = 90 min, adsorbent dosage = 5 mg), indicating that the active sites of CPP200 may have become fully occupied.

#### 3.3.3. Adsorption Kinetics

Two typical adsorption kinetics models, a pseudo-first-order model and a pseudo-second-order model, were used to investigate the adsorption kinetics (Figure S1, Table S1-Supplementary Material). For the adsorption of MB by CPP200, the linear pseudo-second-order model provided an extremely high coefficient of determination (*R*^2^, 0.9994) and an accurate calculated *q_e_* value (*q_e,cal_* = 58.10 mg/g) that was very close to the experimental data (*q_e,exp_* = 59.72 mg/g). In contrast, the pseudo-first-order model yielded inferior results; the difference between the experimental data (*q_e,exp_* = 59.72 mg/g) and the calculated results (*q_e,cal_* = 40.27 mg/g) was obvious. The experimental data of CPP200 for MB were better fitted using the linear pseudo-second-order model, assuming that chemisorption through the sharing or exchange of electrons between adsorbent and adsorbate was the rate-controlling step, and the adsorption rate simultaneously depended on the dosage of adsorbent and the concentration of adsorbate [43].

#### 3.3.4. Adsorption Isotherms

Four adsorption isothermal models, the linear/nonlinear Langmuir and Freundlich equations, were used to study the adsorption of MB by CPP200 (Figure S2, Table S2). The higher coefficient of determination (*R*^2^) showed that the linear Langmuir model was more suitable for describing the adsorption process than the others. As initial MB concentration was increased to higher values (≥50 mg/L), the adsorption capacity (*q_e_*) of CPP200 remained nearly unchanged at a certain temperature, indicating that monolayer adsorption behavior at the CPP200 interface occurred, which was consistent with the Langmuir isothermal model. We could propose that MB was mainly adsorbed on the adsorbent surface in the form of single-layer coverage, but there was no migration of MB onto the CPP200 surface plane [44].

#### 3.3.5. Adsorption Thermodynamics

The effect of temperature on the adsorption of MB by CPP200 was studied by measuring thermodynamic parameters (Figure S3). The standard enthalpy (△*H^θ^*, kJ/mol), standard entropy (△*S^θ^*, J/K/mol) and Gibbs free energy (△*G^θ^*, kJ/mol) [45] calculated values are listed in Table S3. The negative values of △*G^θ^* illustrated the spontaneous nature of MB adsorption onto CPP200. The positive value of △*H^θ^* showed that the adsorption of MB was endothermic, which was consistent with previously discussed results (Figure S2) [46]. Usually, the value of △*H^θ^* can be used to reveal the nature of an adsorption process. The △*H^θ^* value (19.6 kJ/mol) fell within the range of 1–40 kJ/mol, indicating that physical adsorption via electrostatic attraction occurred at all testing temperatures [47]. The positive value of △*S^θ^* indicated that the randomness at the solid–liquid interphase increased during the adsorption process.

#### 3.3.6. Effect of CPP200 Dosage

The identifiable sites and active surface areas could be promoted by increasing the amounts of adsorbent, which greatly affects the adsorption process. Herein, the effect of adsorbent dosage on adsorption was investigated in order to evaluate the influence of this key factor (Figure 7D). With the amount of CPP200 increased from 5.0 mg to 30.0 mg, the MB removal rate increased from 27.53% to 97.43%, respectively. MB removal was expected to increase at higher adsorbent dosages due to increased numbers of active binding sites as well as increased effective surface area [48]. However, the removal rate of MB was not significantly affected by further increasing adsorbent dosages (for example, ≥25.0 mg), indicating that the relatively lower residual MB in the solution would be less likely to interact with CPP200 because of the competitive adsorption between water and MB. Although the removal rate of MB increased significantly, the adsorption capacity of CPP200 decreased from 55.51 mg/g to 32.74 mg/g. We propose that insufficient utilization of the adsorbent occurred, and the active sites were not fully used. Considering the relationship between adsorbent consumption and removal efficiency, 5 mg of adsorbent was used for the following adsorption experiments.

#### 3.3.7. Effect of Solution pH

An important parameter, solution pH value, could affect the surface charge of adsorbent/adsorbate and interfacial transport phenomena [49]. The removal of MB by CPP200 at different pHs was studied (Figure 7E). Meanwhile, the zeta potentials of CPP200 in the pH range of 3.0~10.0 were measured in order to provide information about the surface charge of the adsorbent (Figure 7F). In the pH value range of 3~6, the zeta potential decreased from 13.167 to −38.833, respectively, indicating that an increase in surface electronegativity of BC would facilitate its adsorption capacity for cationic species. Under strong acidic conditions, CPP200 became protonated, which would repel cationic MB, resulting in lower adsorption capacities [50]. In addition, H^+^ and cationic MB would compete for the active sites on the surface of CPP200 [51]. Therefore, the adsorption capacities of CPP200 were relatively lower at lower pH. The electronegativity of CPP200 reached its lowest value at pH = 6.30, while its maximum adsorption capacity could be obtained. In the pH range of 6~8, the zeta potential of CPP200 increased from −38.833 to −15.233, respectively, and the electrostatic attraction between CPP200 and MB gradually weakened, resulting in a gradual decrease in the substrate’s adsorption capacity. At higher pH (>8.34), the surface electronegativity of CPP200 increased again due to the introduced OH^−^, and interactions with cationic MB through electrostatic attraction became stronger. Therefore, the removal rate of MB by CPP200 increased again at high pH [52]. It is worth noting that the pH of aqueous MB solution is 7.32, and CPP200 exhibited slightly lower adsorption capacity for MB at this basicity than the maximum value obtained for it at a pH of 6.30 (Figure 7E). Considering the convenience of experimental operation and the reduced use of toxic reagents, aqueous MB solutions prepared by dissolving MB in deionized water without adjusting pH were directly used for the following adsorption experiments.

### 3.4. Adsorption of Various Dyes on CPP200

**Figure 8:**
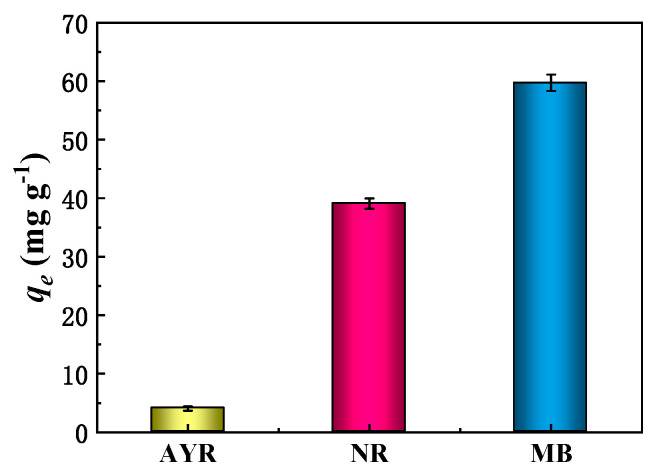
Adsorption capacity of CPP200 toward different dyes in aqueous solutions (dye concentration of 50 mg/L, adsorbent dosage of 5.0 mg and contact time of 90 min, T = 25 °C).

The adsorption capacities of CPP200 toward MB, AYR and NR and their diverse electrical properties were evaluated. Since MB, AYR and NR possess different molecular weights, using units of mmol/g is more suitable for describing and comparing the adsorption capacities of various organic dyes (Figure 8, Table S4). The adsorption capacities of dyes by CPP200 followed the order of AYR (0.0131 mmol/g) < NR (0.1353 mmol/g) < MB (0.1597 mmol/g). It is well known that MB is a cationic dye, while AYR is an anionic dye and NR is a neutral dye. The great difference of adsorption capacities could be attributed to the distinct electrostatic interactions between adsorbate and adsorbent. Therefore, CPP-derived BCs exhibited high adsorption selectivity for cationic dyes.

FT-IR analyses indicated that CPP200 possessed abundant oxygen-containing functional groups such as carboxyl, carbonyl and phenolic species. Thus, the surfaces of BCs were negative charged, facilitating their electrostatic attraction toward cationic dyes, and the adsorption capacity of MB by CPP200 was relatively higher than that for the other tested dyes. Conversely, the electrostatic repulsion between anionic AYR and CPP200 was attributed to the lower adsorption capacity observed.

**Figure 9:**
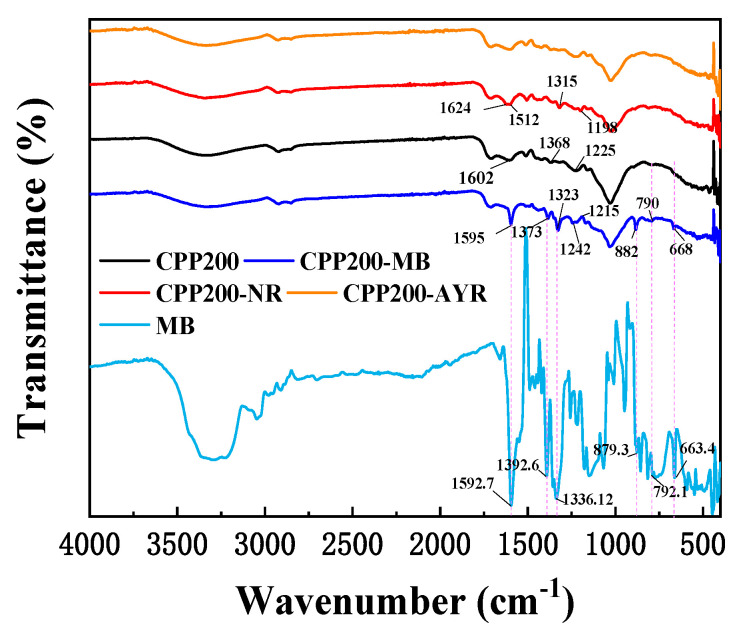
FT−IR spectra of CPP200 pre-and post-adsorption of MB, AYR and NR.

CPP200 post-adsorptions of various organic dyes were analyzed using FT-IR (Figure 9). Following MB adsorption, the FT-IR spectra of CPP200-MB showed weakened bands, suggesting that the oxygen-containing functional groups on the surface of CPP200 were involved in the adsorption process. The oxygen-containing functional groups, such as hydroxyl (–OH), epoxy (–O–), carbonyl (–C=O) and carboxyl (–COOH), could interact with organic substances by forming hydrogen bonds [53]. The hydrogen bonds that were formed between BCs and MB also contributed significantly to the adsorption [54]. Obviously, the peak at 1601.8 cm^−1^ that corresponded to the stretching vibration of –C=O red-shifted to 1595 cm^−1^, and became more pronounced due to hydrogen bonding interactions. In addition, MB could form hydrogen bonds with –COOH and –OH of CPP200, leading to peak broadening and distortions in the wave number range of 3100~3435 cm^−1^. After adsorption of MB, several new peaks appeared at 1373 cm^−1^, 1323 cm^−1^, 1215cm^−1^, 882 cm^−1^, 790 cm^−1^ and 668 cm^−^^1^. The two peaks at 1322.8 cm^−1^ and 1372.8 cm^−^^1^ may be attributed to C–N stretching vibrations [55,56]. The new peak at 882 cm^−^^1^ corresponds to vibrations of C–S bonds, while the new peaks at 789.8 cm^−1^ and 668 cm^−1^ correspond to C–H out-plane bending vibrations for the aromatic rings of MB molecules. The shift of the FT-IR peaks as well as the appearance of new peaks confirmed that MB was successfully adsorbed onto CPP200. For the adsorption of NR by CPP200, the peaks at 1602 cm^−^^1^ and 1225 cm^−1^ red-shifted to 1512 cm^−1^ and 1198 cm^−1^, respectively. Newly-appearing C-N stretching vibrations (1315 cm^−1^) and N=C stretching vibrations (1624 cm^−1^) could be observed in the spectra. Furthermore, we observed that there was basically no change in the spectra of CPP200 both pre- and post-adsorption of AYR. The relatively lower adsorption capacity of CPP200 for AYR may be attributed to electrostatic repulsions between the adsorbate and adsorbent.

### 3.5. Regeneration of CPP200

**Figure 10:**
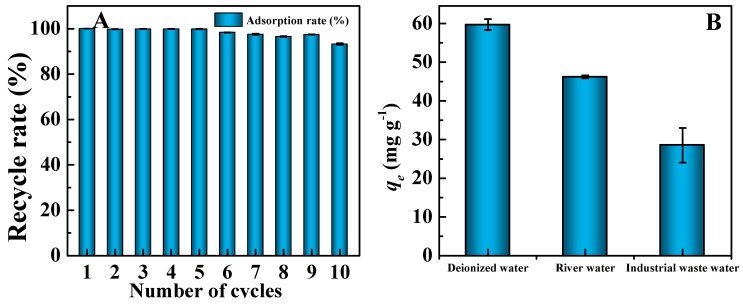
(**A**) Recycle rate of CPP200 for MB in successive 10 adsorption−desorption cycles (*C*_MB_ = 10 mg/L, adsorbent dosage of 50.0 mg and contact time of 90 min, T = 25 °C). (**B**) The adsorption of MB from actual water samples by CPP200 (MB concentration of 50 mg/L, adsorbent dosage of 5.0 mg and contact time of 90 min, T = 25 °C).

The recyclability of an absorbent is very important for its practical application, especially in terms of cost minimization. Therefore, the reusability of CPP200 in the adsorption/desorption of MB was studied (Figure 10A). A quantity of 50.0 mg of CPP200 was shaken in 20.0 mL of MB (10 mg/L) at 25 °C for 90 min under oscillatory conditions, and MB was desorbed from the CPP200-MB using 40.0 mL of HCl (0.1 mol/L). CPP200 could be recovered by suction filtration, after which another adsorption was conducted using the recycled CPP200. The above operations were repeated 10 times (Figure 1B). In the first cycle, the removal rate reached 100%. Furthermore, we could clearly see that even after nine cycles of adsorption/desorption, the removal rate of CPP200 for MB still exceeded 95%. It is worth noting that the removal rates exceeded 99% during the first five cycles, and the removal rate remained high (93.27%) after the tenth cycle of adsorption/desorption. The hydrogen bonding and electrostatic interactions between CPP200 and MB were damaged under acidic conditions, hence the adsorption capacity of CPP200 could be restored [57]. The reutilization results confirmed the excellent regenerative characteristics of CPP200 as well as demonstrated its potential for applications in wastewater treatment. CPP200 may be used as a green, sustainable, highly efficient and recyclable adsorbent for cationic organic dyes.

### 3.6. Adsorption of MB in Actual Water Samples

In order to evaluate the adsorption performance of CPP-derived BCs for MB, different water samples such as deionized water, industrial wastewater and lake water of Yudai River (Changsha, China) were used to prepare 50 mg/L MB solutions (Figure 10B). The adsorption capacity of CPP200 for MB solution prepared by lake water was 46.21 mg/g, which decreased to 28.54 mg/g for MB solution prepared by industrial wastewater. The decrease in adsorption capacity might be attributed to the existence of some metal cations (such as Zn^2+^, Pb^2+^, Fe^3+^, Ca^2+^, etc.) in those water samples. The industrial wastewater tested in this experiment was provided by a local metallurgical plant, which mainly contained some inorganic metal ions such as Zn^2+^ and Pb^2+^. In addition, the industrial wastewater was more acidic (pH = 3.73), which competitively inhibited electrostatic interactions between CPP200 and MB.

### 3.7. Adsorption Mechanisms

**Figure 11:**
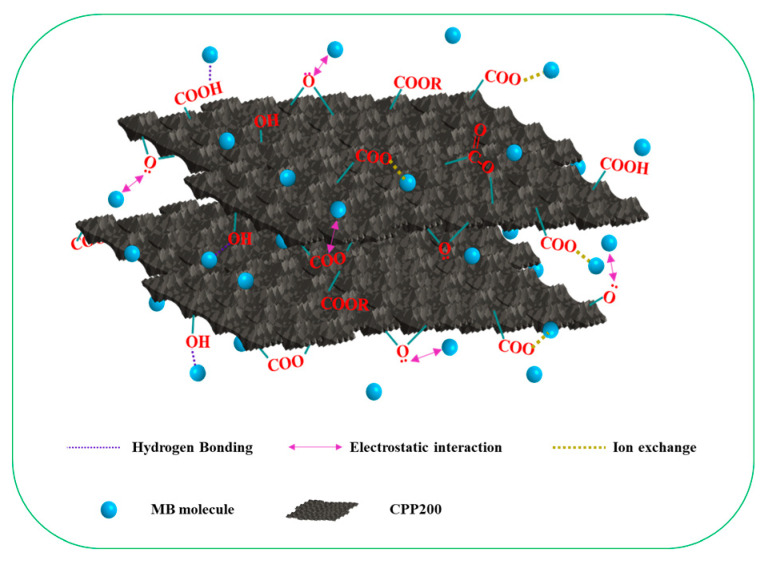
A proposed mechanism for MB adsorption by CPP200.

The adsorption mechanism of BCs toward MB was proposed (Figure 11). MB, a cationic organic dye, contains aromatic and heterocyclic rings. The N and S atoms, as well as its cationic characteristics, greatly affected the interactions between MB and BCs. The adsorption of MB by CPP-derived BCs was achieved through electrostatic interactions, hydrogen bonding, ion exchange and π-π stacking interactions [58]. FT-IR analyses of CPP200 pre- and after-adsorption showed characteristic peaks of MB at 881.6 cm^−1^ (C–S stretching vibrations), at 1372.8 cm^−^^1^ and 1322.8 cm^−1^ (C–N stretching vibrations) and at 789.8 cm^−^^1^ and 667.9 cm^−1^ (in-plane bending vibrations of aromatic hydrocarbons). Furthermore, EDS confirmed the N and S contents in CPP200-MB.

Previous research reported that the adsorption of MB by activated carbon was seriously affected by the electrostatic interactions between the adsorbent and adsorbate [59]. An effect of pH on the adsorption process indicated that electrostatic interactions dominated the adsorption of MB by CPP200. Under low pyrolysis temperatures (≦350 °C), the adsorption capacities of BCs toward MB were affected by the oxygen-containing functional groups which were responsible for the negative charges of BCs [60]. In addition, hydrogen bonds between MB and different oxygen-containing functional groups such as -COOH and –OH in BCs that were prepared at low temperatures were formed [61,62]. The intermolecular π-π interactions may be caused by the π electrons and the aromatic/heterocyclic rings of MB and CPP200. Obviously, the π-π interactions tended to increase with an increase in pyrolysis temperature [63]. As a result of stronger electrostatic interactions, hydrogen bonding and ion exchange dominated the adsorption capacity of CPP-derived BCs, the π-π interactions could consequently be ignored or excluded [64].

### 3.8. Comparisons with Other Adsorbents

As shown in Table 2, a comparison of the maximum MB adsorption capacity of CPP200 with those of other previously reported adsorbents was carried out. Obviously, the CPP-based adsorbents were prepared under relatively mild and non-toxic environmentally friendly conditions. In addition, CPP200 demonstrated relatively excellent adsorption selectivity in addition to its high adsorption capacity.

## 4. Conclusions

Low-temperature, flash-vacuum pyrolysis of an agro-waste, crisp persimmon peel, was performed. CPP200 prepared at 200 °C was negatively charged due to its oxygen-containing groups, which could efficiently interact with positively charged cationic dye (MB) to achieve selective adsorption. Under optimized experimental conditions (initial concentration of MB = 60 mg/L, T ≥ 313 K, time = 90 min, adsorbent dosage = 5 mg), the maximum adsorption capacity of CPP200 reached 77.11 mg/g. The adsorption of MB by CPP200 was spontaneous and endothermic, which could be best described by a linear pseudo-second-order kinetic model as well as a linear Langmuir isothermal model. SEM, elemental mapping, FT-IR spectroscopy, XPS, zeta potential and SSA analyses were used to characterize the physicochemical properties of CPP200 and propose its adsorption mechanism. The experimental data indicated that oxygen-containing groups such as –C=O, –COOH and –OH groups dominated the adsorption of MB by CPP200. The low-temperature, flash vacuum pyrolysis method has shown energy-saving and environmentally-friendly features. Obviously, CPP-derived BCs can be rapidly and conveniently produced, and can thus be used as cost-saving adsorbents for future industrial and practical applications.

## Figures and Tables

**Table 1:** Elemental compositions (at.%) of BC samples prepared by vacuum pyrolysis and CPP200 post-adsorption of MB.

BCs	C	O	S	Mg	K	Ca	N
CPP200	61.1~71.1	25.5~37.4	0.200~0.320	0.260	0.510	0.280	-
CPP250				0.0900	0.990	0.170	-
CPP300				0.150	2.38	0.330	-
CPP350				0.220	1.28	0.250	-
CPP400	78.3~85.3	11.3~15.6	0.320~0.550	0.110	5.01	0.200	-
CPP450				0.150	2.40	0.220	-
CPP500				0.0400	2.72	0.0300	-
CPP200-MB	62.3	36.5	0.810	0.0400	-	0.0100	0.100

**Table 2:** A comparison of adsorption capacities of CPP200 for MB with other previously reported carbon-based adsorbents under optimal experimental conditions.

Adsorbent	Activated Method/ Activator	Temp. (K)	Adsorbent Dosage	C_MB_ (mg/L)	*q*_max_ (mg/g)	References
Persimmon fruit peel	Furnace (700 °C, N_2_)	298	2.0 g/L	1600	303	[18]
Dragon fruit peel	KOH/Furnace (700 °C, N_2_)	323	0.08 g/0.1 L	200	175.0	[62]
Apricot stones	NaOH and Citric acid/Oven (120 °C, N_2_)	298	0. 15 g/0.05 L	160	18.95	[65]
Pulp and Paper Sludge	ZnCl_2_/Tube furnace (700 °C, N_2_)	298	0.01 g/0.01 L	1000	590.20	[66]
Corn cob	H_3_PO_4_/Microwave oven	312.9	0.1 g/0.1 L	34.1	183.3	[67]
Corn stalk	KOH and H_3_PO4/Muffle furnace (700 °C)	298	0.015 g/0.03 L	200	372.3, 105	[68]
Crisp persimmon peel	Vacuum tube furnace (200 °C)	313	0.005 g/0.02 L	60	77.11	This study

## Data Availability

Not applicable.

## Supplementary Materials

The following supporting information can be downloaded at: https://www.mdpi.com/article/10.3390/molecules27165160/s1, Figure S1: Fitted adsorption kinetic curves by pseudo-first-order models: (A) Linear form; (B) Non-linear form. Fitted adsorption kinetic curves by pseudo-second-order models: (C) Linear form; (D) Non-linear form; Figure S2: The experimental data of CPP200 toward MB and the fitting curves of Langmuir isotherm model: (A) Linear form; (B) Non-linear form. The experimental data of CPP200 toward MB and the fitting curves of Freundlich isotherm model: (C) Linear form; (D) Non-linear form; Figure S3: Experimental data and the fitted curve of In*k_d_* versus 1/T calculated from van’t Hoff plots of adsorption of MB onto CPP200; Table S1: Adsorption kinetic parameters for the adsorption of MB onto CPP200; Video S1: title; Table S2: Adsorption isothermal parameters for the adsorption of MB onto CPP200; Table S3: Thermodynamic parameters of MB adsorption onto CPP200; Table S4: Molecular structure, molecular weight and maximum absorbance of dyes.

## References

[1] Raees A., Jamal M.A., Ahmed I., Silanpaa M., Saad Algarni T. (2021). Synthesis and characterization of CeO_2_/CuO nanocomposites for photocatalytic degradation of methylene blue in visible light. Coatings.

[2] Shooto N.D., Thabede P.M., Bhila B., Moloto H., Naidoo E.B. (2020). Lead ions and methylene blue dye removal from aqueous solution by mucuna beans (velvet beans) adsorbents. J. Environ. Chem. Eng..

[3] Mandal B., Panda J., Paul P.K., Sarkar R., Tudu B. (2020). MnFe_2_O_4_ decorated reduced graphene oxide heterostructures: Nanophotocatalyst for methylene blue dye degradation. Vacuum.

[4] He J., Du Y.-E., Bai Y., An J., Cai X., Chen Y., Wang P., Yang X., Feng Q. (2019). Facile Formation of Anatase/Rutile TiO_2_ Nanocomposites with Enhanced Photocatalytic Activity. Molecules.

[5] Yang J.-Y., Jiang X.-Y., Jiao F.-P., Yu J.-G. (2018). The oxygen-rich pentaerythritol modified multi-walled carbon nanotube as an efficient adsorbent for aqueous removal of alizarin yellow R and alizarin red S. Appl. Surf. Sci..

[6] Silva L.G.M., Moreira F.C., Cechinel M.A.P., Mazur L.P., de Souza A.A.U., Souza S.M.A.G.U., Boaventura R.A.R., Vilar V.J.P. (2020). Integration of Fenton’s reaction based processes and cation exchange processes in textile wastewater treatment as a strategy for water reuse. J. Environ. Manag..

[7] Turkay O., Barışçı S., Dimoglo A. (2016). Kinetics and mechanism of methylene blue removal by electrosynthesized ferrate (VI). Sep. Sci. Technol..

[8] Chennah A., Anfar Z., Amaterz E., Taoufyq A., Bakiz B., Bazzi L., Guinneton F., Benlhachemi A. (2020). Ultrasound-assisted electro-oxidation of Methylene blue dye using new Zn_3_(PO_4_)_2_ based electrode prepared by electro-deposition. Mater. Today Proc..

[9] Mosavi S.A., Ghadi A., Gharbani P., Mehrizad A. (2021). Photocatalytic removal of Methylene Blue using Ag@CdSe/Zeoilte nanocomposite under visible light irradiation by Response Surface Methodology. Mater. Chem. Phys..

[10] Azari A., Nabizadeh R., Nasseri S., Mahvi A.H., Mesdaghinia A.R. (2020). Comprehensive systematic review and meta-analysis of dyes adsorption by carbon-based adsorbent materials: Classification and analysis of last decade studies. Chemosphere.

[11] Uddin M.J., Ampiaw R.E., Lee W. (2021). Adsorptive removal of dyes from wastewater using a metal-organic framework: A review. Chemosphere.

[12] Qin F., Li J., Zhang C., Zeng G., Huang D., Tan X., Qin D., Tan H. (2022). Biochar in the 21st century: A data-driven visualization of collaboration, frontier identification, and future trend. Sci. Total Environ..

[13] Liu S., Luo X., Xing Y., Tan S., Jiang Y., Huang Q., Chen W. (2022). Natural bioaugmentation enhances the application potential of biochar for Cd remediation. Sep. Purif. Technol..

[14] Bagotia N., Sharma A.K., Kumar S. (2021). A review on modified sugarcane bagasse biosorbent for removal of dyes. Chemosphere.

[15] You X., Wang R., Zhu Y., Sui W., Cheng D. (2021). Comparison of adsorption properties of a cellulose-rich modified rice husk for the removal of methylene blue and aluminum (III) from their aqueous solution. Ind. Crop. Prod..

[16] Todescato D., Mayer D.A., Cechinel M.A.P., Hackbarth F.V., de Souza A.A.U., de Souza S.M.A.G.U., Vilar V.J.P. (2021). Cork granules as electron donor in integrated reduction/oxidation and sorption processes for hexavalent chromium removal from synthetic aqueous solution. J. Environ. Chem. Eng..

[17] Porto B., Goncalves A.L., Esteves A.F., de Souza S.M.A.G.U., de Souza A.A.U., Vilar V.J.P., Pires J.C.M. (2021). Assessing the potential of microalgae for nutrients removal from a landfill leachate using an innovative tubular photobioreactor. Chem. Eng. J..

[18] Ates A., Oymak T. (2020). Characterization of persimmon fruit peel and its biochar for removal of methylene blue from aqueous solutions: Thermodynamic, kinetic and isotherm studies. Int. J. Phytoremediat..

[19] Odinga E.S., Waigi M.G., Gudda F.O., Wang J., Yang B., Hu X., Li S., Gao Y. (2020). Occurrence, formation, environmental fate and risks of environmentally persistent free radicals in biochars. Environ. Int..

[20] Sazykin I.S., Minkina T.M., Khmelevtsova L.E., Antonenko E.M., Azhogina T.N., Dudnikova T.S., Sushkova S.N., Klimova M.V., Karchava S.K., Seliverstova E.Y. (2021). Polycyclic aromatic hydrocarbons, antibiotic resistance genes, toxicity in the exposed to anthropogenic pressure soils of the Southern Russia. Environ. Res..

[21] Chen X., Yang L., Myneni S.C.B., Deng Y. (2019). Leaching of polycyclic aromatic hydrocarbons (PAHs) from sewage sludge-derived biochar. Chem. Eng. J..

[22] Zeghioud H., Fryda L., Djelal H., Assadi A., Kane A. (2022). A comprehensive review of biochar in removal of organic pollutants from wastewater: Characterization, toxicity, activation/functionalization and influencing treatment factors. J. Water Process Eng..

[23] Long L., Sun S., Zhong S., Dai W., Liu J., Song W. (2010). Using vacuum pyrolysis and mechanical processing for recycling waste printed circuit boards. J. Hazard. Mater..

[24] Zhuang Q.-Q., Cao J.-P., Wu Y., Zhao M., Zhao X.-Y., Zhao Y.-P., Bai H.-C. (2021). Heteroatom nitrogen and oxygen co-doped three-dimensional honeycomb porous carbons for methylene blue efficient removal. Appl. Surf. Sci..

[25] Matheus J.R.V., de Andrade C.J., Miyahira R.F., Fai A.E.C. (2020). Persimmon (*Diospyros kaki* L.): Chemical properties, bioactive compounds and potential use in the development of new products—A review. Food Rev. Int..

[26] Testoni A., Bellini E., Giordani E. (2002). In Post-harvest and processing of persimmon fruit. First Mediterranean Symposium on Persimmon.

[27] Wang Z., Gao M., Li X., Ning J., Zhou Z., Li G. (2020). Efficient adsorption of methylene blue from aqueous solution by graphene oxide modified persimmon tannins. Mater. Sci. Eng. C.

[28] Shen Z., Jin F., Wang F., McMillan O., Al-Tabbaa A. (2015). Sorption of lead by Salisbury biochar produced from British broadleaf hardwood. Bioresour. Technol..

[29] Sima N.A.K.K., Ahmad S.T., Pessarakli M. (2013). Comparative study of different salts (sodium chloride, sodium sulfate, potassium chloride, and potassium sulfate) on growth of forage species. J. Plant Nutr..

[30] Ying Z., Chen X., Li H., Liu X., Zhang C., Zhang J., Yi G. (2021). Efficient Adsorption of Methylene Blue by Porous Biochar Derived from Soybean Dreg Using a One-Pot Synthesis Method. Molecules.

[31] El-Azazy M., El-Shafie A.S., Morsy H. (2021). Biochar of Spent Coffee Grounds as Per Se and Impregnated with TiO_2_: Promising Waste-Derived Adsorbents for Balofloxacin. Molecules.

[32] Zapata-Hernandez C., Durango-Giraldo G., Cacua K., Buitrago-Sierra R. (2020). Influence of graphene oxide synthesis methods on the electrical conductivity of cotton/graphene oxide composites. J. Text. Inst..

[33] Huang W., Chen J., Zhang J. (2020). Removal of ciprofloxacin from aqueous solution by rabbit manure biochar. Environ. Technol..

[34] Zong P., Jiang Y., Tian Y., Li J., Yuan M., Ji Y., Chen M., Li D., Qiao Y. (2020). Pyrolysis behavior and product distributions of biomass six group components: Starch, cellulose, hemicellulose, lignin, protein and oil. Energy Convers. Manag..

[35] Wei Y., Shen C., Xie J., Bu Q. (2020). Study on reaction mechanism of superior bamboo biochar catalyst production by molten alkali carbonates pyrolysis and its application for cellulose hydrolysis. Sci. Total Environ..

[36] Abdoul Magid A.S.I., Islam M.S., Chen Y., Weng L., Li J., Ma J., Li Y. (2021). Enhanced adsorption of polystyrene nanoplastics (PSNPs) onto oxidized corncob biochar with high pyrolysis temperature. Sci. Total Environ..

[37] Fernandes B.C.C., Mendes K.F., Dias A.F., da Silva Caldeira V.P., da Silva Teofilo T.M., Severo Silva T., Mendonca V., de Freitas Souza M., Valadao Silva D. (2020). Impact of Pyrolysis Temperature on the Properties of Eucalyptus Wood-Derived Biochar. Materials.

[38] Din S.U., Awan J.M., Imran M., Zain Ul A., Haq S., Hafeez M., Hussain S., Khan M.S. (2021). Novel nanocomposite of biochar-zerovalent copper for lead adsorption. Microsc. Res. Tech..

[39] Liu S., Li J., Xu S., Wang M., Zhang Y., Xue X. (2019). A modified method for enhancing adsorption capability of banana pseudostem biochar towards methylene blue at low temperature. Bioresour. Technol..

[40] Hoslett J., Ghazal H., Mohamad N., Jouhara H. (2020). Removal of methylene blue from aqueous solutions by biochar prepared from the pyrolysis of mixed municipal discarded material. Sci. Total Environ..

[41] Cornelissen G., Gustafsson O., Bucheli T.D., Jonker M.T.O., Koelmans A.A., Van Noort P.M. (2005). Extensive Sorption of Organic Compounds to Black Carbon, Coal, and Kerogen in Sediments and Soils Mechanisms and Consequences for Distribution, Bioaccumulation, and Biodegradation. Environ. Sci. Technol..

[42] Kumari S., Chauhan G.S., Ahn J.H. (2016). Novel cellulose nanowhiskers-based polyurethane foam for rapid and persistent removal of methylene blue from its aqueous solutions. Chem. Eng. J..

[43] Jiang L., Wen Y., Zhu Z., Liu X., Shao W. (2021). A Double cross-linked strategy to construct graphene aerogels with highly efficient methylene blue adsorption performance. Chemosphere.

[44] Dubey S., Gusain D., Sharma Y.C. (2016). Kinetic and isotherm parameter determination for the removal of chromium from aqueous solutions by nanoalumina, a nanoadsorbent. J. Mol. Liq..

[45] McKay G., Mesdaghinia A., Nasseri S., Hadi M., Aminabad M.S. (2014). Optimum isotherms of dyes sorption by activated carbon: Fractional theoretical capacity & error analysis. Chem. Eng. J..

[46] Mallakpour S., Tabesh F. (2019). Tragacanth gum based hydrogel nanocomposites for the adsorption of methylene blue: Comparison of linear and non-linear forms of different adsorption isotherm and kinetics models. Int. J. Biol. Macromol..

[47] Egbosiuba T.C., Abdulkareem A.S., Kovo A.S., Afolabi E.A., Tijani J.O., Auta M., Roos W.D. (2020). Ultrasonic enhanced adsorption of methylene blue onto the optimized surface area of activated carbon: Adsorption isotherm, kinetics and thermodynamics. Chem. Eng. Res. Des..

[48] Jawad A.H., Rashid R.A., Ishak M.A.M., Wilson L.D. (2016). Adsorption of methylene blue onto activated carbon developed from biomass waste by H_2_SO_4_ activation: Kinetic, equilibrium and thermodynamic studies. Desalination Water Treat..

[49] Crini G., Peindy H., Gimbert F., Robert C. (2007). Removal of C.I. Basic Green 4 (Malachite Green) from aqueous solutions by adsorption using cyclodextrin-based adsorbent: Kinetic and equilibrium studies. Sep. Purif. Technol..

[50] Ghorai S., Sarkar A., Raoufi M., Panda A.B., Schonherr H., Pal S. (2014). Enhanced removal of methylene blue and methyl violet dyes from aqueous solution using a nanocomposite of hydrolyzed polyacrylamide grafted xanthan gum and incorporated nanosilica. ACS Appl. Mater. Interfaces.

[51] Foo K.Y., Hameed B.H. (2011). Microwave assisted preparation of activated carbon from pomelo skin for the removal of anionic and cationic dyes. Chem. Eng. J..

[52] El-Bouraie M. (2014). Removal of the Malachite Green (MG) Dye from Textile Industrial Wastewater Using the Polyurethane Foam Functionalized with Salicylate. J. Disper. Sci. Technol..

[53] Hu B., Ai Y., Jin J., Hayat T., Alsaedi A., Zhuang L., Wang X. (2020). Efficient elimination of organic and inorganic pollutants by biochar and biochar-based materials. Biochar.

[54] Jawad A.H., Abdulhameed A.S., Mastuli M.S. (2020). Acid-factionalized biomass material for methylene blue dye removal: A comprehensive adsorption and mechanism study. J. Taibah Univ. Sci..

[55] Xiong L., Yang Y., Mai J., Sun W., Zhang C., Wei D., Chen Q., Ni J. (2010). Adsorption behavior of methylene blue onto titanate nanotubes. Chem. Eng. J..

[56] Ai L., Zhang C., Liao F., Wang Y., Li M., Meng L., Jiang J. (2011). Removal of methylene blue from aqueous solution with magnetite loaded multi-wall carbon nanotube: Kinetic, isotherm and mechanism analysis. J. Hazard. Mater..

[57] Chang Z., Chen Y., Tang S., Yang J., Chen Y., Chen S., Li P., Yang Z. (2020). Construction of chitosan/polyacrylate/graphene oxide composite physical hydrogel by semi-dissolution/acidification/sol-gel transition method and its simultaneous cationic and anionic dye adsorption properties. Carbohydr. Polym..

[58] Dassanayake R.S., Acharya S., Abidi N. (2021). Recent Advances in Biopolymer-Based Dye Removal Technologies. Molecules.

[59] Karagoz S., Tay T., Ucar S., Erdem M. (2008). Activated carbons from waste biomass by sulfuric acid activation and their use on methylene blue adsorption. Bioresour. Technol..

[60] Yuan J.-H., Xu R.-K., Zhang H. (2011). The forms of alkalis in the biochar produced from crop residues at different temperatures. Bioresour. Technol..

[61] Wang H., Li J., Ding N., Zeng X., Tang X., Sun Y., Lei T., Lin L. (2020). Eco-friendly polymer nanocomposite hydrogel enhanced by cellulose nanocrystal and graphitic-like carbon nitride nanosheet. Chem. Eng. J..

[62] Jawad A.H., Saud Abdulhameed A., Wilson L.D., Syed-Hassan S.S.A., Alothman Z.A., Rizwan Khan M. (2021). High surface area and mesoporous activated carbon from KOH-activated dragon fruit peels for methylene blue dye adsorption: Optimization and mechanism study. Chin. J. Chem. Eng..

[63] Wei J., Tu C., Yuan G., Liu Y., Bi D., Xiao L., Lu J., Theng B.K.G., Wang H., Zhang L. (2019). Assessing the effect of pyrolysis temperature on the molecular properties and copper sorption capacity of a halophyte biochar. Environ. Pollut..

[64] Chen B., Zhou D., Zhu L. (2008). Transitional adsorption and partition of nonpolar and polar aromatic contaminants by biochars of pine needles with different pyrolytic temperatures. Environ. Sci. Technol..

[65] Kavci E., Erkmen J., Bingöl M.S. (2021). Removal of methylene blue dye from aqueous solution using citric acid modified apricot stone. Chem. Eng. Commun..

[66] Zhao F., Shan R., Li W., Zhang Y., Yuan H., Chen Y. (2021). Synthesis, Characterization, and Dye Removal of ZnCl_2_-Modified Biochar Derived from Pulp and Paper Sludge. ACS Omega.

[67] Jawad A.H., Bardhan M., Islam M.A., Islam M.A., Syed-Hassan S.S.A., Surip S.N., Alothman Z.A., Khan M.R. (2020). Insights into the modeling, characterization and adsorption performance of mesoporous activated carbon from corn cob residue via microwave-assisted H_3_PO_4_ activation. Surf. Interfaces.

[68] Liu L., Li Y., Fan S. (2019). Preparation of KOH and H_3_PO_4_ Modified Biochar and Its Application in Methylene Blue Removal from Aqueous Solution. Processes.

