# Supplementary material for: A Novel Low-Cost Bio-Sorbent Prepared from Crisp Persimmon Peel by Low-Temperature Pyrolysis for Adsorption of Organic Dyes
Source: Molecules. 2022 Aug 12;27(16):5160. doi: 10.3390/molecules27165160 (PMC9416227; doi:10.3390/molecules27165160)
Supplement: Supplementary file 1 [file molecules-27-05160-s001.zip › molecules-1837090-supplementary.pdf]

molecules

MDPI

Article

A Novel Low-Cost Bio-Sorbent Prepared from Crisp Persimmon Peel under Lower Temperature for Selective Adsorption of Methylene Blue

Lu-Qing Xie, Xin-Yu Jiang and Jin-Gang Yu[*]

College of Chemistry and Chemical Engineering, Central South University, Changsha 410083, China
* Correspondence: yujg@csu.edu.cn

1. Adsorption kinetics

Two couples of kinetic models were used to fit the experimental data of contact time of MB adsorbed on the biochar derived from crisp persimmon peel. The first couple of models, linear and nonlinear pseudo-first-order models, could be expressed as follows:

$$\text{Linear equation: } \ln(q_e - q_t) = \ln q_e - k_1 t \quad \text{(S1)}$$

$$\text{Non-linear equation: } q_t = q_e(1\text{-}e^{-k_1 t}) \quad \text{(S2)}$$

The second couple of models, linear and nonlinear pseudo-second-order models, could be expressed as follows:

$$\text{Linear equation: } \frac{t}{q_t} = \frac{1}{k_2 q_e^2} + \frac{t}{q_e} \quad \text{(S3)}$$

$$\text{Non-linear equation: } q_t = \frac{k_2 q_e{}^2 t}{1 + k_2 q_e t} \quad \text{(S4)}$$

where t (min) is the contact time; q_e (mg/g) and q_t (mg/g) are the amounts of MB adsorbed at equilibrium and at time t, respectively; k_1 (min^{-1}) and k_2 (g/(mg·min)) are the specific adsorption rate constants of the pseudo first-order model and the pseudo second order models, respectively.

To whom correspondence should be addressed.

[*] JG Yu, E-mail: yujg@csu.edu.cn; Tel/Fax: +86-731-88879616.

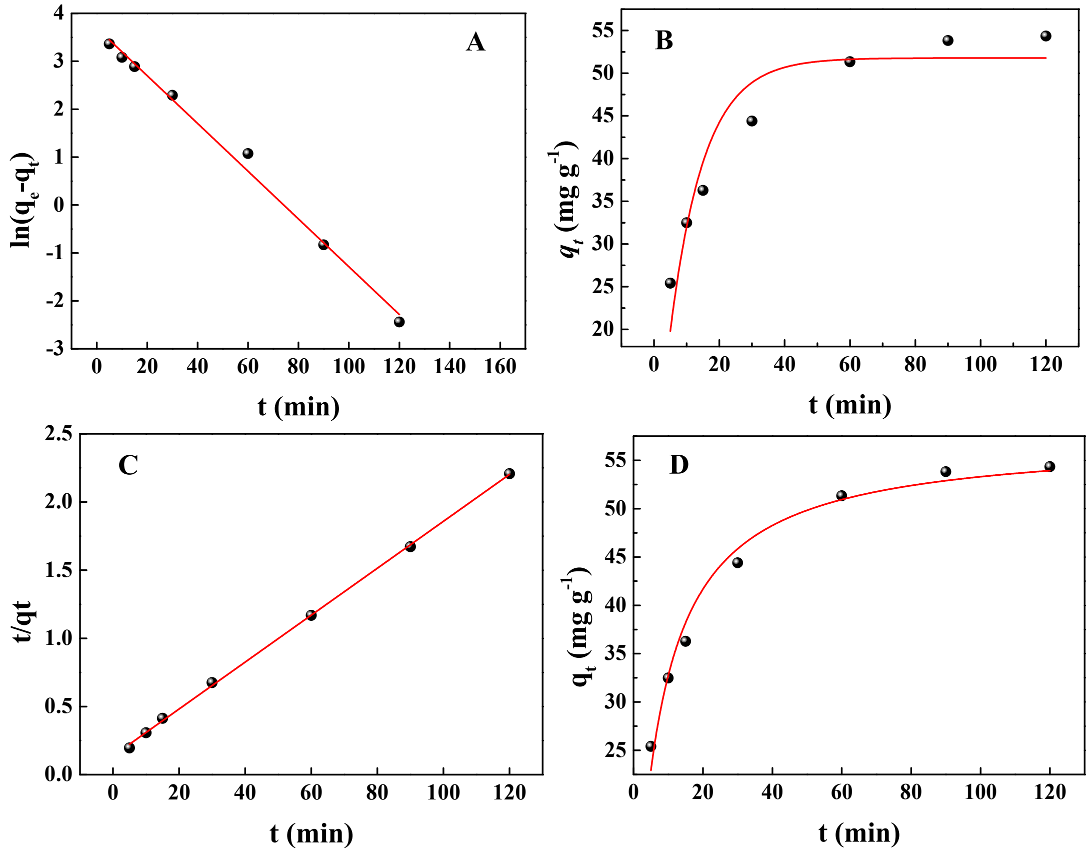

Figure S1. Fitted adsorption kinetic curves by pseudo-first-order models: (**A**) Linear form; (**B**) Non-linear form. Fitted adsorption kinetic curves by pseudo-second-order models: (**C**) Linear form; (**D**) Non-linear form.

Table S1. Adsorption kinetic parameters for the adsorption of MB onto CPP200.

Fitting parameters	Linear pseudo-first-order model	Non-linear pseudo-first-order model	Linear pseudo-second-order model	Non-linear pseudo-second-order model
R^2	0.992	0.887	0.999	0.980
k_1 (min^{-1})	0.0498	0.0963		
k_2 (min^{-1})			0.00215	0.00233
q_e (mg/g)	40.3	51.8	58.1	57.3

2. Adsorption isotherms and thermodynamics

Two thermodynamic models were used to fit the experimental data of MB adsorbed on CPP200.

Linear Langmuir model:

$$\frac{C_e}{q_e} = \frac{C_e}{q_m} + \frac{1}{q_m k_L} \tag{S5}$$

Non-linear Langmuir model:

$$q_e = \frac{q_m k_L C_e}{1 + k_L C_e} \tag{S6}$$

where C_e (mg/L) is the equilibrium concentration; q_e (mg/g) is the amount of adsorbed species per specified amount of adsorbent; K_L is the Langmuir equilibrium constant and q_m (mg/g) is the amount of adsorbate required to form an adsorbed monolayer.

Linear Freundlich isotherm model:

$$\ln q_e = \ln k_F + \frac{1}{n} \ln C_e \quad \text{(S7)}$$

Non-linear Freundlich model:

$$q_e = k_F C_e^{\frac{1}{n}} \quad \text{(S8)}$$

where C_e (mg/L) is the equilibrium concentration of adsorbent; q_e (mg/g) is the amount of adsorbent per unit mass; K_F and n are two Freundlich constants: n represents the relative advantage of adsorption process; K_F is the affinity constant which is related to the adsorption capacity of the adsorbent and can also be defined as adsorption or distribution coefficient, indicating the amount of dye adsorbed on CPP200 at unit equilibrium concentration.

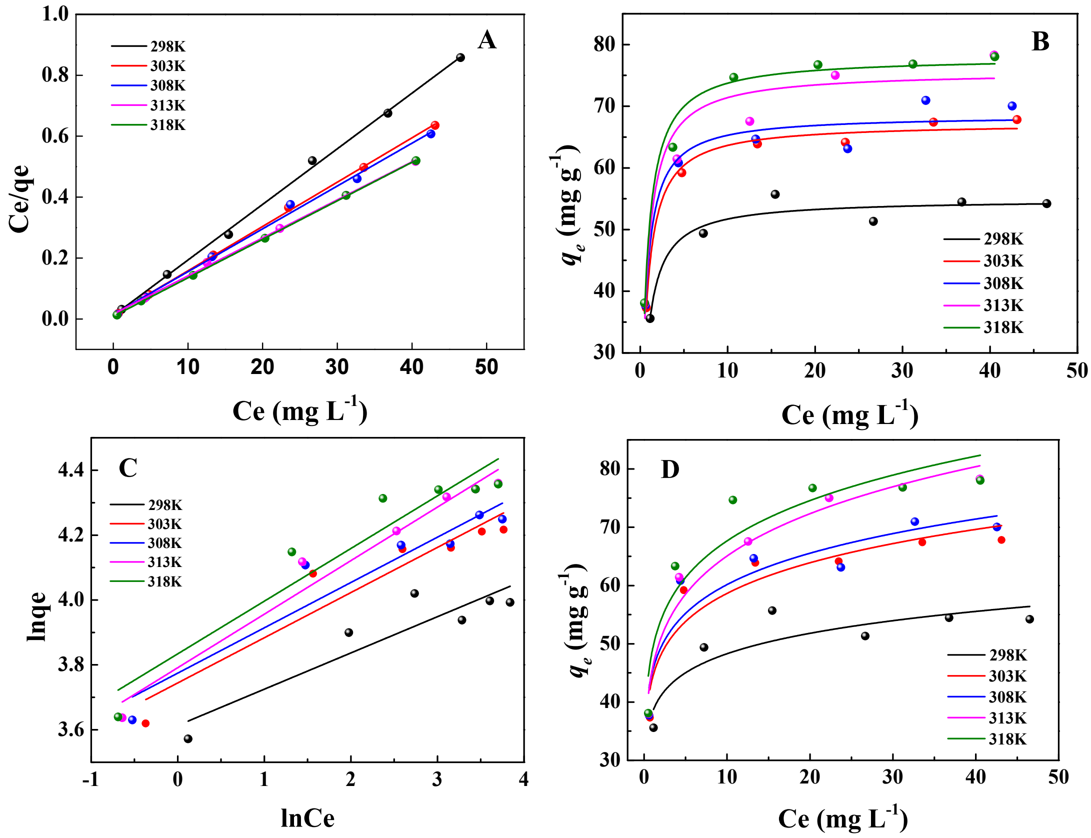

Figure S2. The experimental data of CPP200 toward MB and the fitting curves of Langmuir isotherm model: (**A**) Linear form; (**B**) Non-linear form. The experimental data of CPP200 toward MB and the fitting curves of Freundlich isotherm model: (**C**) Linear form; (**D**) Non-linear form.

Table S2. Adsorption isothermal parameters for the adsorption of MB onto CPP200.

	Model	Fitting parameters	298K	303K	308K	313K	318K
**Linear forms**	Langmuir	R^2	0.998	0.999	0.995	0.998	0.999
		k_L	1.65	1.13	0.939	0.754	1.41
		q_m (mg/g)	54.8	68. 8	71.2	80.1	79.1
	Freundlich	R^2	0.805	0.876	0.881	0.958	0.890
		k_F	37.1	42.3	43.6	44.3	46.3
		n	8.95	7.18	7.16	6.06	6.16
**Non-linear forms**	Langmuir	R^2	0.930	0.986	0.939	0.920	0.976
		k_L	1.62	1.76	2.01	1.69	1.75
		q_m (mg/g)	54.9	67.3	68.5	75.6	78.0
	Freundlich	R^2	0.760	0.863	0.848	0.957	0.867
		k_F	38.3	44.1	45.3	45.8	49.0
		n	9.90	8.08	8.09	6.55	7.12

Thermodynamic parameters including enthalpy (ΔH^θ, kJ/mol), entropy (ΔS^θ, J/(K·mol)) and Gibbs free energy (ΔG^θ, kJ/mol) of the adsorption process can be calculated by **Equations (S9–11).**

$$k_d = \frac{C_0 - C_e}{C_e} \times \frac{V}{m} \tag{S9}$$

$$\ln k_d = \frac{-\Delta G^\theta}{RT} = \frac{\Delta S^\theta}{R} - \frac{\Delta H^\theta}{RT} \tag{S10}$$

$$\Delta G^\theta = \Delta H^\theta - T\Delta S^\theta \tag{S11}$$

where k_d (L/mg) is the thermodynamic equilibrium constant; V (L) and m (g) are the volume of solution and the mass of CPP200, respectively; T (K) is the absolute temperature and R (8.314 J/(mol·K)) is the ideal gas constant.

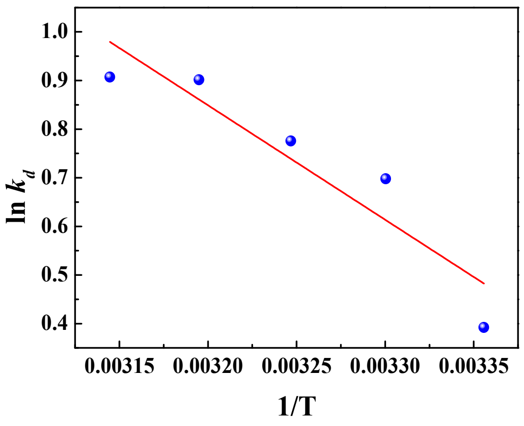

Figure S3. Experimental data and the fitted curve of Ink_d versus 1/T calculated from van't Hoff plots of adsorption of MB onto CPP200.

Table S3. Thermodynamic parameters of MB adsorption onto CPP200.

T (K)	ΔG^θ (kJ/mol)	ΔH^θ (kJ/mol)	ΔS^θ (J/(K·mol))
298K	−1.20		
303K	−1.54		
308K	−1.89	19.6	69.7
313K	−2.24		
318K	−2.59		

3. Properties of dye molecules

Table S4. Molecular structure, molecular weight and maximum absorbance of dyes.

Dyes	MB	NR	AYR
Structural formula	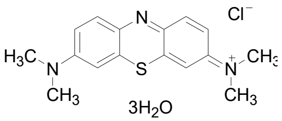	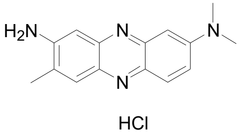	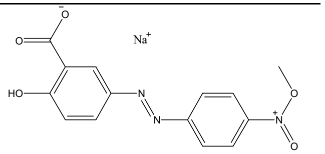
Molecular formula	$C_{16}H_{18}N_3SCl{\cdot}3H_2O$	$C_{15}H_{16}N_4{\cdot}HCl$	$C_{13}H_8N_3O_5Na$
Molecular weight	373.90	288.78	309.22
Maximum absorbance (nm)	664	542	370
